# The relevance of the real-world evidence in research, clinical, and regulatory decision making

**DOI:** 10.3389/fpubh.2025.1512429

**Published:** 2025-02-18

**Authors:** Valton Costa, Marcelo Graziano Custodio, Eran Gefen, Felipe Fregni

**Affiliations:** ^1^Neuromodulation Center and Center for Clinical Research Learning, Spaulding Rehabilitation Hospital, Massachusetts General Hospital, Harvard Medical School, Boston, MA, United States; ^2^Laboratory of Neuroscience and Neurological Rehabilitation, Physical Therapy Department, Federal University of Sao Carlos, São Carlos, Brazil; ^3^Global Innovation and Development, Established Pharmaceuticals Division, Abbott Products Operations AG, Allschwil, Switzerland

**Keywords:** real-world data, real-world evidence, clinical research, regulatory process, decision-making, evidence-based medicine

## Abstract

The scientific method has been established as the optimal approach for systematically gathering and interpreting data on various human phenomena, mainly through the adoption of strict experimental methods, such as controlled randomized trials, which is relevant for clinical decision-making and research, but also weights the regulatory processes for approval of drugs and other medical products. However, a key factor in strict methods is the generalizability of findings that may be limited to specific settings and patient characteristics. This limitation can be addressed by non-experimental methods aimed at investigating populations in naturalistic routine clinical settings, which may offer a more representative reflection of the usage, effectiveness, and safety of healthcare interventions. These approaches can generate the so-called real-world data and the resulting real-world evidence. In this narrative review, we present these concepts, explore the potential applications and advantages of real-world evidence for clinical, research, and regulatory decision-making, and discuss the challenges of employing it, the solutions to improve its generation, and lastly the current level of evidence required for the integration of real-world evidence into regulatory decision-making. We concluded that the advantages of using it, when utilized in a balanced manner, overcome the challenges and, therefore, can offer a time- and cost-saving solution for researchers, the healthcare industry, regulatory agencies, policymakers, and payers towards the patient benefit. The knowledge generated by this approach provides valuable additional insights into medical interventions in real patients under realistic daily scenarios.

## 1 Introduction

The scientific method has long been established as the optimal approach for systematically gathering and interpreting data on various natural phenomena, including human health. Through experimentation and observation, this process transforms raw data into valuable information, which accumulates and forms the basis of evidence. Scientific knowledge plays a crucial role in generating health information and guiding clinical decision-making for the benefit of individuals and communities. This foundation is encapsulated in the concept of Evidence-Based Medicine, which emphasizes the integration of scientific evidence, clinician expertise, and patient preferences in the decision-making process (1). In research, the accuracy of subsequent steps hinges on the quality of data collection. Poor data collection may result in false assumptions, ultimately leading to healthcare malpractices. To mitigate these risks, clinical research has traditionally adhered to a positivist view, aiming to control for predictable biases and measure outcomes as objectively as possible.

Historically, the gold standard for measuring the effects and efficacy of interventions has been the experimental trials with a randomized and controlled design (RCT). RCTs are widely recognized for ensuring rigorous control over experimental conditions, establishing causality between interventions and outcomes, and minimizing bias in comparing treatments (2, 3). However, there exists a trade-off between experimental control and the generalizability of findings. While well-designed and adequately powered RCTs provide accurate results based on systematic data collection and analysis, their generalizability may be limited to specific settings or patient characteristics defined by study protocols. This limitation can fail to capture the heterogeneity of the real-world population and clinical settings in various aspects (2, 4, 5). In everyday clinical practice, patients are not constrained by study protocols or ideal scenarios; they exhibit biological and demographic heterogeneity (6). Therefore, data collected from patients’ every day lives may offer a more representative reflection of the usage, effectiveness, and safety of healthcare interventions.

Although not entirely precise in its terminology, “real-world data” (RWD) refers mainly to data collected in clinical contexts regarding patient health status and healthcare delivery, derived from routine healthcare practices rather than controlled research settings (4, 7). Certainly, all data collected from real people, whether in research settings or daily clinical practice, are RWD. However, this term emphasizes the *sources* and *settings* of data collection, indicating that it originates from subjects in naturalistic situations. This terminology has been widely adopted and consistently used by major regulatory bodies and agencies worldwide (2, 8–10). Consequently, the structured evidence derived from the analysis of this type of data is referred to as “real-world evidence” (RWE).

RWD and the resulting evidence have been extensively utilized by the pharmaceutical industry and developers of healthcare technologies to gain valuable insights into customer desires, needs, and preferences on health products and healthcare services throughout various stages of the product lifecycle. Additionally, RWD is increasingly valuable in other settings, including research on the development of new drugs and healthcare materials, to inform study design and gather insights on the efficacy and safety of health products in real-world settings (see Table 1). RWE can be generated prospectively or retrospectively from observational studies and other sources of RWD. Consequently, regulatory agencies, particularly in high-income countries, where such data is more frequently collected and systematically analyzed, have begun considering RWE in guiding marketing approval and regulatory decisions, as well as monitoring the long-term usage, safety, and effectiveness of drugs and medical technologies (11). In this paper, we aim to explore the potential applications of RWD and RWE, as well as the challenges associated with improving data collection and analysis. We will also examine their utility in clinical decision-making, regulatory purposes, and as a complementary strategy to conventional experimental research for generating supportive evidence.

**Table 1:** Real-world data potential use in healthcare.

Guide early-phase research in the development of new drugs, devices, or therapies Inform internal research and business decisions Facilitate the development of new drugs and healthcare materials Improve the understanding of the impact of a specific therapy on different patient profiles in real-world settings Gain insights into customer desires, needs, and preferences in various stages of product and service lifecycle Generate new evidence Informing study design Refining research designs Collected through various designs Including populations underrepresented in RCTs Being time and cost-saving Involving large datasets, facilitating subgroup analysis Providing insights into how medical treatments and interventions perform in daily life

## 2 Sources of RWD

Sources of RWD encompass healthcare databases such as electronic health records and registries, health insurance systems, wearable and mobile devices, social media, and patient-led network platforms (12, 13). They provide valuable insights into healthcare utilization, treatment efficacy, adverse effects, and the natural history of certain conditions. Healthcare databases may document routine clinical appointments, laboratory tests, and medication prescriptions. Registries, whether local or national, compile extensive data on treatment delivery for specific populations and may maintain biobanks. Examples of such sources include the European Cystic Fibrosis Society registries, the British Society for Rheumatology’s national registry (patients on biologic therapy), the National Cancer Registration and Analysis Service, the Clinical Practice Research Datalink and the QResearch in the UK, and the American Academy of Orthopedic Surgeons registry (orthopedic care) (9). Additionally, administrative databases, particularly in the context of health insurance and pharmacological dispensation, offer crucial information on healthcare costs and the long-term effects of health interventions throughout inpatient and outpatient services, surgeries, hospitalizations, diagnostics, laboratory, and pharmacy services. Medicare and Medicaid in the USA and the Ontario Pharmacy Evidence Network in Canada are examples of such databases, which utilize RWD to optimize health information and service delivery (9).

Furthermore, emerging sources of health-related RWD include social media and social network forums, where patients actively contribute data by sharing their treatment experiences, medication adherence, and reasons for avoiding treatment (13). Patient-driven networks, which may include clinicians and researchers, have also emerged to provide platforms for sharing health-related information and personal treatment experiences. Examples of such networks include PatientsLikeMe and the National Patient-Centered Clinical Research Network in the USA, which supports multiple disease studies, and the Accelerated Cure Project, which shares clinical data and biosamples for multiple sclerosis research.

## 3 Potential uses of RWE

### 3.1 Advantages of RWD

Many of the advantages of RWD studies stem from the limitations of RCTs. RWD encompasses data collected from groups that are often underrepresented in research due to either ethical, costs, timelines, or strategic considerations, such as children, adolescents, pregnant women, the older adult, and individuals with multiple comorbidities and vulnerabilities (4, 14, 15). It provides insights into how medical interventions perform in daily life scenarios, capturing the complexities and variations in effectiveness encountered in routine clinical practice. These variations may include the challenges faced by healthcare professionals, such as different treatment regimens, patient adherence, duration of follow-up, healthcare delivery dynamics, and resource availability (6). RWD studies often involve larger datasets than RCTs, facilitating subgroup analysis, modeling, and the generalizability of findings (15, 16). Moreover, RWD studies complement traditional evidence from RCTs by uncovering information that may not be attainable through more controlled methods (see Table 2) (3, 17). This approach directly benefits patients by addressing unmet needs often overlooked in conventional healthcare delivery, improving understanding of off-label applications, and identifying the risks and limitations of products and services for community health—all in a more time-efficient manner.

**Table 2:** The advantages and challenges of utilizing RWE.

Advantages
Reduce drug development costs Complement traditional evidence Avoid the exposition of patients to unnecessary trials Allow hybrid methodologies, combining aspects of RCTs and observational studies Guide marketing approval and regulatory decisions Use for regulatory purposes (accelerate regulatory approval, extend drug-approved indications, add/change safety information in drug leaflets, revoke drug usage) Optimize approval processes and timelines Monitor the long-term usage, safety, and effectiveness of medical devices, drugs, and healthcare technologies Provide substantial elements to drive clinical decisions toward the patient’s benefit Optimize health resource allocation Complement clinical decision-making beyond the controlled experimental settings

Challenges
Data sources lack the rigor and standardization observed in RCTs Existence of incomplete, inconsistent, or varying-quality data, compromising the reliability and accuracy of study findings Biases and confounding factors may be introduced, undermining internal validity Controlling biases and confounders requires sophisticated statistical techniques and careful consideration of study design and analytical methods Complex and time-consuming data source integration Restricting regulation on data privacy and security

RWD has been extensively utilized in post-marketing approval pharmacovigilance to monitor long-term drug safety and efficacy. For instance, the USA Food and Drug Administration (FDA) does this through the Sentinel Initiative System (18). Observational studies offer a significant advantage in conducting safety evaluations of drugs and other health products. These studies often utilize retrospective data collected from electronic health records, disease registries, and claim databases, ensuring that data collection is not influenced by the *a priori* objectives of the study. Furthermore, RWD can be collected through various observational designs, e.g., cohort studies, surveys, and case–control, either prospectively or retrospectively. Alternatively, RWD can be collected in the context of pragmatic clinical trials, which emphasize the naturalistic behavior of patients in using a given treatment. Pragmatic trials might encompass relevant population groups, experienced investigators, standard care as control, and outcomes relevant to the population under study (15, 19). Conversely, retrospective analysis offers a time-saving alternative to the lengthy trial phases of drug testing, providing urgent responses to gaps in the healthcare system. Although retrospective analysis can offer reliable insights into the safety of medications in conditions or subgroups lacking RCT-based evidence, insights need to be interpreted with caution in cases where a given event cannot be compared to a control group (13, 16, 20).

Moreover, RWD can provide valuable insights for guiding early-phase research in the development of medical products. Some sources can contribute patient data for integrating an external comparator in single-arm trials or exploring various clinical outcomes, such as long-term and quality-of-life outcomes and adverse events, which are often overlooked in RCTs. Leveraging RWD in this manner can improve care standards, support epidemiological research, expedite public health policies, and save time and resources (2, 4, 21, 22). Insights from RWD analysis can inform internal research and business decisions within the healthcare industry, guide payers, and facilitate cost analysis (23, 24).

### 3.2 Challenges of using RWD and possible solutions

RWD often lacks the rigor and standardization observed in RCTs. This results in inconsistent data, compromising the reliability of findings. Confounding factors may be introduced, undermining internal validity. Addressing these issues requires improved data collection, completeness, and consistency (13). Likewise, integrating diverse sources of RWD, such as electronic health records and administrative databases, is complex and time-consuming due to variations in data formats and coding systems. Lack of standardization and interoperability hinders data aggregation and analysis (see Table 2). Efforts to standardize data collection practices and improve interoperability can enhance the usefulness of RWD (12, 25).

RWD studies are susceptible to selection bias and confounding factors due to the non-randomized nature of data collection in real-world settings. Factors such as patient demographics, healthcare-seeking behavior, and provider preferences may influence the likelihood of certain individuals being included in analyses, potentially skewing study results and conclusions. Controlling for these factors requires sophisticated statistical techniques, consideration of study design, and analytical methods. Some advanced techniques, such as propensity score matching, inverse probability weighting, stratification for the generation of statistically comparable controls, sensitivity analyses, and language processing tools, can be useful as it has been used previously with relative success (4, 26–29). Creating interconnected networks between research centers and pharmaceutical and biomedical companies can contribute to the quality and accuracy of RWD. They can collectively store and analyze larger cohort data and share tools and insights for accurate data analysis, which can be integrated into subsequent study designs (30).

RWD often contains sensitive patient information, raising concerns about data privacy and security. Safeguarding patient privacy and complying with data protection regulations, such as the Health Insurance Portability and Accountability Act in the USA and the General Data Protection Regulation in the European Union, present significant challenges for researchers and organizations handling RWD. It may raise the complexity level for the development of strategic therapies for the entire population based on restrictions on data access. However, ensuring adequate technical solutions for data anonymization, encryption, and access controls is essential to mitigate the risk of unauthorized breaches and protect patient confidentiality.

Compliance with study design guidelines and ethical principles is essential to maintain the integrity of RWD studies and protect the rights and welfare of research participants. A few recommendations may help to improve the utilization of RWD and the generation of evidence: (i) improvement of electronic data recordings for research purposes; (ii) enhancing completeness, consistency, and accuracy of data collection and storage (10); (iii) improvement of the interoperability and exchange of electronic health information, facilitating data utilization for different stakeholders; (iv) planning of rigorous observational designs, identification of appropriate databases and proper study registration (28); and (v) transparent and complete reporting of studies, especially after marketing approval of health products, which can be achieved by adopting known guidelines, such as the STROBE for observational studies and the CONSORT for pragmatic trials, potentially improving reporting and validity (16, 31).

## 4 Regulatory standards for the use of RWE

Regulatory bodies and stakeholders, including legislative actors, have integrated RWE into regulatory decisions concerning medical devices, drugs, and health materials. This integration necessitates rigorous analytic methodology to establish the efficacy and safety of these products. Consequently, regulatory bodies and research centers, particularly in the USA, Europe, and some parts of the world, have taken action to establish optimal utilization of this evidence for regulatory purposes. They have produced guidelines and initiatives to generate standards of practice in using RWE (2, 10, 32–34). However, there is still disagreement regarding the stage at which it should be incorporated (35). Some advocate for its use after marketing approval for surveillance purposes, while others present cases of its use in both pre- and post-approval stages, for instance, to regulate orphan drugs. Instances of drug approval without RCTs by the European Medicine Agency (EMA) and FDA have been reported (10, 16, 36).

The integration of RWE into the regulatory process remains a topic of debate. While some argue for the sufficiency of RWE to support the efficiency and safety of health products, most literature emphasizes its use as a complement to RCTs (4, 9, 12, 35). Before RCTs, it can generate hypotheses to be further investigated in stricter designs aiming to establish efficacy. After RCTs, it holds the potential for evaluating the transferability of novel treatments and findings to broader populations, facilitating external validity. Independently, RWE can be used to assess long-term efficacy, safety, and other outcomes of interest, especially when RCTs are not feasible (28).

The design of studies with hybrid methodologies, combining aspects of RCTs and observational studies, offers promise. For example, using pragmatic trial approaches and external controls in single-arm trials can merge practical aspects with statistical rigor. How does this impact the regulatory process? There is a growing trend in regulatory drug approval processes to embrace RWE in decision-making. This is evident in cases from various parts of the world where RWE has been used to accelerate regulatory approval, extend drug-approved indications, add safety information to drug labels, revoke drug usage, or implement changes. Regulatory agencies and pharmaceutical companies have shown openness to utilizing RWE to find accelerated solutions to meet health system needs, particularly in the development, approval, effectiveness, and safety evaluation of drugs for broader or specific populations, combining RCT results with further evidence from RWD analysis beyond Phase IV trials (2, 21, 36–39).

## 5 Level of evidence from RWD studies

In scientific research, evidence is hierarchically classified based on ranking systems that assess the reliability, validity, and applicability of evidence from different study designs. While classification systems may vary, they generally prioritize designs with better internal validity, bias control, and the ability to establish causality between interventions and outcomes. According to common evidence evaluation systems, RCTs and systematic reviews based on valid RCTs are on top of the hierarchy (level 1 and 2 evidence), while observational studies, case–control studies, and studies with external controls fall at level 4, positioned between non-randomized controlled cohorts (level 3) and expert opinion and case reports (level 5) (1, 40). Interestingly, a systematic review of nested case–control studies or an observational study with dramatic effects for common harms is considered at the highest hierarchical level (40).

This hierarchical view of evidence guides decisions made in clinical settings for patient treatment, resource allocation, and research implementation, though current works in evidence hierarchy are aware of the complexity of this view. Nonetheless, it is an integral aspect of market authorization of new medical products, serving to prevent ineffective or harmful products from entering the market (41). Decisions are made based on the expected and available evidence and may be subject to regulatory standards about the type and quantity of research supporting the authorization application. Historically, market authorization has predominantly relied on RCTs, particularly for assessing drug effectiveness (42). Therefore, incorporating other study designs into the process presents challenges, as it may be perceived to reduce the reliability of evidence. Regulatory agencies, such FDA and EMA are increasingly highlighting the relevance of RWE in their decision-making processes, complementing evidence from other sources, and supporting pre-authorization and post-approval assessments (8, 18, 34, 42).

Therefore, if different study designs are to be considered solely or in combination with traditional RCTs, they need to be part of an equally reliable process, that is, with a similar level of evidence and the same rates of false positive and false negative cases—the proportion of products that will be granted a license or approval but have not the intended clinical effect or disapproved but the intended benefit—expected for the conventional use of RCT evidence to approve a drug or medical product (41). While there is a willingness to integrate RWE into marketing approval and decision-making, there are inconsistent perceptions regarding what constitutes adequate levels of evidence among regulators and stakeholders. Observational studies face challenges in determining the strength of evidence, particularly in controlling bias, especially in prospective designs. However, we do not have evidence to support the notion that, for relevant outcomes such as treatment effects and adverse events, there are robust differences between RCTs and well-designed observational studies in terms of overestimation and quality of the results, though specific observational designs like case–control may tend to overestimate harm as compared to cohort and cross-sectional studies (43–46).

Thus, an important question is how many studies and what kind of RWD study are necessary, with an emphasis on the quality of the primary data and the expected methodology that must be deemed as convincing evidence for regulatory and licensing purposes. The answer to that question may vary depending on circumstances, but it is agreed that decisions should ideally be based on multiple and cumulative sources of evidence, encompassing experimental and observational studies, retrospective and prospective designs, and pragmatic considerations (2, 20, 41). As new types and sources of RWD become widely available—mostly in the form of big data—regulatory agencies, policymakers, clinicians, and researchers must collaborate to establish standards for regulatory decisions, determining the quantity and quality of studies deemed as high-level evidence.

## 6 Conclusion

The understanding of real-world evidence has gained traction in recent years, with an increasing number of possible uses, from healthcare product development and approval to the final clinical decision and guidance of public policies. The advantages of RWE use, when utilized in a balanced manner, overcome the challenges and, therefore, can offer a time- and cost-saving solution for researchers, the healthcare industry, regulatory agencies, policymakers, and payers towards the patient benefit. Future research should explore strategies to integrate diverse RWD sources to build robust databases that enhance utility and generalizability for stakeholders. Promising methodologies, such as artificial intelligence-powered approaches to ensure validity, hybrid research designs, and large-scale longitudinal and multicenter RWD cohorts, could significantly contribute to the development and credibility of this type of evidence.
